# Interleukin-18 and COVID-19

**DOI:** 10.1017/S0950268821002636

**Published:** 2021-12-16

**Authors:** C. M. Schooling, M. Li, S. L. Au Yeung

**Affiliations:** 1 Li Ka Shing Faculty of Medicine, School of Public Health, The University of Hong Kong, Hong Kong SAR, China; 2 City University of New York, Graduate School of Public Health and Health Policy, New York, NY, USA

**Keywords:** COVID-19, evolutionary biology, interleukin-18, Mendelian randomisation

## Abstract

Vulnerability to coronavirus disease (COVID)-19 varies due to differences in interferon gamma (IFN*γ*) immunity. We investigated whether a key modifiable interferon precursor, interleukin-18, was related to COVID-19, overall and by severity, using Mendelian randomisation. We used four established genome-wide significant genetic predictors of interleukin-18 applied to the most recent genome-wide association study of COVID-19 (June 2021) to obtain Mendelian randomisation inverse variance weighted estimates by severity, i.e. any (cases = 112 612, non-cases = 2 474 079), hospitalised (cases = 24 274, non-cases = 2 061 529) and very severe (cases = 8779, non-cases = 1 001 875) COVID-19. To be comprehensive, we also conducted an exploratory analysis for IFN*γ* and two related cytokines with less well-established genetic predictors, i.e. interleukin-12 and interleukin-23. Genetically predicted interleukin-18 was associated with lower risk of any COVID-19 (odds ratio (OR) 0.96 per standard deviation, 95% confidence interval (0.94–0.99, *P*-value 0.004)) and of very severe COVID-19 (OR 0.88, 95% CI 0.78–0.999, *P*-value 0.048). Sensitivity analysis and a more liberal genetic instrument selection gave largely similar results. Few genome-wide significant genetic predictors were available for IFN*γ*, interleukin-12 or interleukin-23, and no associations with COVID-19 were evident. Interleukin-18 could be a modifiable target to prevent COVID-19 and should be further explored in an experimental design.

Vulnerability to coronavirus disease (COVID)-19 varies, partly because of structurally determined differences in exposure to severe acute respiratory syndrome coronavirus 2 (SARS-CoV-2) [1], undoubtedly most effectively addressed by equitable public health prevention and control policies. However, some differences in vulnerability to COVID-19 may also be determined by physiological factors, such as differences in immune function, whose elucidation could further inform prevention and treatment.

The human immune system has been shaped globally and locally by pathogens over millennia, given the importance of surviving to have children [2], resulting in environmentally determined time and place-specific vulnerability to infections [3]. Currently, evidence for any particular immune system cytokine affecting vulnerability to COVID-19 is limited [4, 5], although modulating interleukin 6 can be an effective treatment [6]. However, a genetic difference that impairs interferon gamma (IFN*γ*) immune response resulting in unusually severe disease on encountering specific infections, such as the mycobacteria causing tuberculosis [3, 7, 8], has also recently been found relevant to COVID-19 [7, 9]. IFN*γ* was also related to the risk of severe acute respiratory syndrome (SARS) in 2003 [10, 11]. Correspondingly, functional analysis of SARS-CoV-2 proteins suggests the virus is vulnerable to IFN*γ* [12]. Taken together, these studies suggest that IFN*γ* or its precursors, such as interleukin-18 [13], interleukin-12 or interleukin-23, could have a role in COVID-19. Interleukin-18 has been suggested as a target of intervention in COVID-19 [14, 15]. Notably, the role of interleukin-18 in immune function is also substantiated by its role in several auto-immune diseases (inflammatory bowel disease and eczema/dermatitis) [16, 17].

Here, we used a two-sample Mendelian randomisation (MR) study design, where possible. MR uses genetic proxies of exposures to reduce confounding by taking advantage of the random allocation of genetic material at conception [18]. A two-sample MR design has the advantage of being able to assess the effects of exposure on outcome even when no sample including both exposure and outcome exists, but requires genetic proxies of the exposures. Given only established genetic predictors of interleukin-18 exist [16, 17], we primarily assessed the role of interleukin-18 in COVID-19 by severity, and secondarily the role of interleukin-12, interleukin-23 and IFN*γ*.

## Methods

This is a two-sample MR study, i.e. an instrumental variable analysis with genetic instruments using separate samples for the primary exposure of interleukin-18, if possible, for IFN*γ*, but only completely overlapping samples for interleukin-12 and −23. We used strong (*P* < 5 × 10^−8^), independent (*r*^2^ < 0.001) genetic predictors of each exposure ideally as previously used or else derived them from an existing genome-wide association study (GWAS). We also only used genetic predictors with an *F*-statistic >10, obtained using an established approximation [19]. Where associations were found we also repeated the analysis using a more liberal cut-off of genetic predictors, i.e. *P* < 5 × 10^−6^. We sought correlated proxies (*r*^2^ > 0.8) from ldlink (https://ldlink.nci.nih.gov/) for any genetic predictors not available in the most recent GWAS of COVID-19. We used number of children as a positive control outcome for interleukin-18, because given the selective pressure on vulnerability to infections [2, 3], protective immune function differences would be expected to have disbenefits for fertility [2].

### Data sources

#### Genetic predictors of interleukin-18

Genetic predictors of interleukin-18 (effect sizes) were taken from a previous study [16, 17] where they were obtained from a GWAS of 3636 Finnish individuals from The Cardiovascular Risk in Young Finns Study (mean age men 37.4 years, women 37.5%) and FINRISK2002 (mean age men 60.4, women 60.1%) adjusted for age, sex, body mass index and the first 10 genetic principal components [20]. These genetic predictors explained about 7% of the variance in interleukin-18 [17].

#### Genetic predictors of interleukin-12, interleukin-23 and interferon gamma

Genetic predictors of interleukin-12, interleukin-23 and IFN*γ* were obtained from proteomic GWAS of the INTERVAL study in 3301 participants (mean age 43.7 years, 49.9% women) of European ancestry [21] and/or FINNRISK [20]. Genetic associations from INTERVAL were adjusted for age, sex, duration between blood draw and processing and the first three principal components of ancestry from multi-dimensional scaling [21].

#### Genetic associations with COVID-19

We obtained genetic associations with COVID-19 from the latest publicly available GWAS summary statistics (https://www.covid19hg.org/results/r6/) (accessed 18 June 2021), comparing genetic make-up for different severities of COVID-19 with the population, i.e. very severe COVID-19 (cases = 8779, non-cases = 1 001 875), hospitalised COVID-19 (cases = 24 274, non-cases = 2 061 529) and any COVID-19 (cases = 112 612, non-cases = 2 474 079). Very severe COVID-19 was hospitalisation with laboratory-confirmed SARS-CoV-2 infection based on RNA and/or serology and hospitalisation with COVID-19 as the primary reason for admission, followed by death or respiratory support. Case status for hospitalised COVID-19 was hospitalised with laboratory-confirmed infection, hospitalisation due to COVID-19-related symptoms or self-reported hospitalised COVID-19-positive. Case status for any COVID-19 was laboratory-confirmed SARS-CoV-2 infection (RNA and/or serology-based), physician diagnosis of COVID-19 or self-report as COVID-19-positive. The COVID-19 GWAS is mainly based on people of European descent from existing cohort studies and was adjusted for study covariates, principal components, age, sex, age^2^ and sex × age, as appropriate (https://www.covid19hg.org/results/r6/).

#### Genetic associations with number of children

We obtained genetic associations with responses to the questions ‘How many children have you fathered?’ in men (*n* = 209 872) and ‘How many children have you given birth to? (Please include live births only)’ in women (*n* = 250 782) reported at recruitment to the UK Biobank taken from publicly available genetic summary statistics [22] pertaining to Europeans adjusted for genotype array and 10 principal components. The UK Biobank is a cohort study of half a million people intended to be aged 40–69 years (average age 57 years) at recruitment in 2006–2010 in England, Wales and Scotland [23].

### Statistical analysis

We aligned genetic variants on the same effect allele for each analysis, dropping palindromic genetic variants where effect allele frequency was not given for the exposure. We sought highly correlated proxies, from ldlink (https://ldlink.nci.nih.gov/), for genetic variants available for an exposure but not an outcome. We obtained MR estimates using inverse variance weighting (IVW) meta-analysis of the Wald estimates (genetic variant on outcome divided by genetic variant on exposure) with multiplicative random effects for >3 genetic instruments [24]. The weighted median and MR-Egger with different assumptions for validity were used as sensitivity analysis [25]. The weighted median is valid as long as >50% of the weight comes from valid instruments [25]. The MR-Egger intercept provides an indication of the validity of the IVW estimate assuming the genetic instruments do not affect confounders of exposure on outcome [25], but has limited interpretability when the number of instruments is low because it is based on fitting a line to genetic instruments on outcome against genetic instruments on exposure.

We estimated power using the approximation that the sample size for an MR study is the sample size for exposure on outcome divided by the *r*^2^ for instruments on exposure [26], obtained using maximum likelihood [27]. Given the genetic variants for interleukin-18 explain 7% of the variance [17], this study has sufficient power to detect odds ratios of about 0.97, 0.94 and 0.88 for any, hospitalised and very severe COVID-19, respectively, per standard deviation of interleukin-18. Analysis of publicly available data does not require ethical approval. For the analysis, we used R 4.1.2 [28], the R packages MendelianRandomization to obtain estimates and metafor to meta-analyse them and MR-Base to extract and harmonise instruments [29].

## Results

We used the four established independent genome-wide significant genetic variants for interleukin-18 (rs385076 (*NLRC4*), rs17229943 (*OCLN*), rs71478720 (*IL10*), rs115267715 (*CDK7*)) (Supplementary Table S1) [16, 17]. The *F*-statistics were >10, mean 30.7. None of these variants were palindromic. rs115267715 was not available for very severe COVID-19 and had no close correlate with *r*^2^ > 0.80, the *r*^2^ for the closest correlate was 0.65. Of the three available GWAS of interleukin-12 in the INTERVAL study, only one GWAS had an independent genome-wide significant variant, i.e. rs7208047 (*RPL7P48*) (Supplementary Table S1). Of the three available GWAS of interleukin-23 from the INTERVAL study, one GWAS had two independent genome-wide significant variants (rs9815073 (*LPP*) and rs4921223 (*AC008697.1*)) (Supplementary Table S1). Of the two available GWAS of IFN*γ*, the GWAS from the INTERVAL study had two genome-wide significant variants (rs7459901 (*RP11-756K15.2*) and rs7567468 (*UGT1A4*)) (Supplementary Table S1).

Interleukin-18 was inversely associated with any COVID-19 and very severe COVID-19 using IVW, with a directionally similar estimate for hospitalised COVID-19 (Table 1). Sensitivity analysis estimates were similar using the weighted median but not using MR-Egger (Table 1). Using a more liberal selection of genetic variants (*P*-value <5 × 10^−6^) interleukin-18 remained associated with any COVID-19 using IVW; the weighted median and MR-Egger estimates were similar (Table 1). Given a more liberal selection of genetic variants may be more likely to violate the exclusion-restriction assumption, we also used MR-robust adjusted profile score (RAPS) to assess over-dispersion [30]. However, MR-RAPS did not indicate any over-dispersion for the more liberal selection of genetic variants; the simple model gave very similar estimates to IVW (Table 1). Interleukin-18 was also associated with fewer children (Supplementary Table S2).

**Table 1.** Mendelian randomisation estimates for genetically predicted interleukin-18 (standard deviation) [16, 17] on different severities of COVID-19 in the largest available GWAS largely of people of European descent compared to a population sample in the COVID19-hg GWAS meta-analysis round 6 (https://www.covid19hg.org) using different methods with both genome-wide and liberal instrument selection

Cut-off	SNP #	COVID-19 severity	Method	Odds ratio	95% confidence interval	*P*-value	MR-Egger	
							Intercept *P*-value	*Q* statistic (*P*-value)
Genome-wide significant (5 × 10^−8^)	3	Very severe	IVW	0.88	0.78–0.999	0.047		
			WM	0.95	0.82–1.11	0.57		
			MRE	3.43	0.49–24.2	0.21	0.17	1.74 (0.19)
	4	Hospitalised	IVW	0.95	0.89–1.01	0.09		
			WM	0.95	0.88–1.02	0.17		
			MRE	1.39	0.82–2.37	0.23	0.16	0.31 (0.86)
	4	Any	IVW	0.97	0.94–0.99	0.004		
			WM	0.96	0.94–0.99	0.007		
			MRE	1.06	0.85–1.33	0.58	0.39	0.62 (0.73)
Liberal	13	Very severe	IVW	0.96	0.89–1.04	0.30		
(5 × 10^−6^)			WM	1.00	0.91–1.11	0.99		
			MRE	1.06	0.93–1.21	0.37	0.08	11.4 (0.41)
			MR-RAPs	0.96	0.89–1.03	0.26		
	16	Hospitalised	IVW	0.96	0.92–1.01	0.11		
			WM	0.97	0.92–1.03	0.29		
			MRE	1.03	0.95–1.11	0.53	0.08	17.0 (0.25)
			MR-RAPs	0.96	0.92–1.001	0.055		
	16	Any	IVW	0.98	0.97–1.00	0.03		
			WM	0.98	0.96–1.00	0.02		
			MRE	0.98	0.96–1.01	0.25	0.98	9.81 (0.78)
			MR-RAPs	0.98	0.97–1.00	0.03		

SNP, single nucleotide polymorphism; IVW, inverse variance weighted; WM, weighted median; MRE, MR-Egger; MR-RAPs, MR robust adjusted profile score using the simple model.

Genetic predictors for interleukin-12, interleukin-23 and IFN*γ* all had *F*-statistics >10, but were not associated with any type of COVID-19 (Supplementary Table S3).

## Discussion

Consistent with previous studies showing impaired interferon response to infection associated with COVID-19 [3, 7–9] and functional analysis suggesting the virus is vulnerable to IFN*γ* [12], we show that a key IFN*γ* precursor, interleukin-18, is associated with a lower risk of any COVID-19 and possibly of very severe COVID-19.

Interleukin-18 is thought to play a broad role in defence against infections [15]. Observational evidence concerning the role of interleukin-18 in reducing the risk of COVID-19 is limited [31, 32] and difficult to interpret because it concerns observational studies in patients where interleukin-18 could represent a protective response, a symptom or a cause of complications. Currently, to our knowledge, no experimental evidence concerning the role of interleukin-18 in COVID-19 exists. However, experimental evidence exists concerning the role of interleukin-18 in other viruses. Specifically, interleukin-18 was shown to protect mice against death from herpes simplex 1 [33], possibly via natural killer cells [34, 35] or via IFN*γ* [33]. Interleukin-18 has also been shown to protect mice against murine coronavirus mouse hepatitis virus strain A59 by preventing viral replication via IFN*γ*, although interleukin-1 was not similarly protective [36]. Interleukin-18 has also been shown to offer some protection against rotavirus in mice, possibly via caspase-inducing apoptosis [37], and so has been suggested as a potential means of addressing emerging and recalcitrant viruses [37]. More broadly, interleukin-18 protecting against infection is consistent with it increasing the risk of some auto-immune and atopic conditions [16, 17], and also accords with the well-established theory that reproduction trades-off against survival, as interleukin-18 also reduced the number of children (Supplementary Table S2).

Despite these coherent findings, this study has limitations. First, we checked the MR assumptions of relevance, independence and exclusion restriction from the data available, but observational confirmation of causal relations gives reassurance but is not definitive. To address relevance, we primarily only used genome-wide significant predictors, which have been used before to identify the effects of interleukin-18 [16, 17]. The underlying GWAS all addressed the possibility of confounding by population stratification. Exclusion restriction was addressed by considering the possibility of selection bias from only recruiting survivors [38], and sensitivity analysis with different assumptions. Interleukin-18 was not associated with survival (based on paternal attained age in a study of over a million lifespans) [39] (Supplementary Table S4), making bias from selective survival of genetic make-up and competing risk of COVID-19 less likely. Sensitivity analysis generally gave similar estimates, although MR-Egger estimates using genome-wide significant genetic predictors of interleukin-18 differed from the other estimates perhaps because the small number of instruments makes the MR-Egger estimate obtained from a line through three or four points difficult to interpret, this discrepancy was less evident using a larger number of instruments (Table 1). This study is limited by the design of the underlying studies. The COVID-19 GWAS largely represents cases with symptomatic disease, which is of most concern to population health. Given we took advantage of publicly available summary genetic associations, it was not possible to assess whether the associations are non-linear, or to assess differences by sex, which could be informative given COVID-19 risk and immune system functions differ by sex [40]. We did not examine the role of other linked elements of the immune system, such as interleukin-12p70 because its genetic predictors form a tightly correlated cluster with vascular endothelial growth factor, interleukin-13 and interleukin-10 [20], so would be unlikely to predict only interleukin-12p70. Finally, we could not examine all the relations of interest in detail, because all the underlying GWAS used to generate instruments were relatively small. In addition, only MR estimates for interleukin-18 were obtained using separate samples. Instruments for interleukin-12 and −23, and for IFN*γ* could only be obtained from GWAS included in the COVID-19 GWAS, possibly biasing towards the confounded estimates. The most reliable evidence likely concerns interleukin-18 which had the most extensive and validated instruments.

Despite these limitations, this study has some consistency with a recent MR study searching systematically for COVID-19 druggable targets that homed in on drugs targeting *ACE2* and *IFNAR2* or *IL10RB* [41]. More generally, it is also consistent with COVID-19 representing a failure of the immune system to mount an effective immune response which might then generate an over-reaction. Currently, interleukin-18 inhibitors are being investigated as a means of preventing auto-immune diseases [42, 43]; however, this study suggests that like other immunosuppressant biologics [44], they might have adverse effects on some specific infections. Whether promoting interleukin-18 or its consequences, such as IFN*γ*, might be helpful to prevent or treat COVID-19 could be considered, given these treatments are available [45, 46], and a small case series suggested promising results of using IFN*γ* in critically ill COVID-19 patients [46]. In this context, given differences in immune response to COVID-19, including for interleukin-18 [47], it might also be worth considering whether the use of these interventions should differ by sex. Finally, this study suggests that conditions, such as eczema, related to interleukin 18 may be associated with greater resilience to COVID-19.

## Conclusion

Interleukin-18 might protect against COVID-19. COVID-19 may partially represent an initial failure of the immune system. Whether any such failure could be prevented by recombinant interleukin-18 might be considered.

## Data Availability

All data used in this study are publicly available.

## Supplementary material

For supplementary material accompanying this paper visit https://doi.org/10.1017/S0950268821002636. click here to view supplementary material

## Author statement

C.M.S. had full access to the data and takes full responsibility for the results.
