# Supplementary material for: Interleukin-18 and COVID-19
Source: Epidemiol Infect. 2021 Dec 16;150:e14. doi: 10.1017/S0950268821002636 (PMC8755530; doi:10.1017/S0950268821002636)
Supplement: Supplementary file 1 [file S0950268821002636sup001.docx]

Supplementary Table 1: Details of the genome wide significant genetic variants used to predict interleukin-18, interleukin-12, interleukin-23 and interferon-γ

| Exposure | SNP | Gene | chr | position | Effect allele | Other allele | beta | p-value | Standard error |
| --- | --- | --- | --- | --- | --- | --- | --- | --- | --- |
| IL-18 | rs115267715 | *CDK7* | 5 | 68535015 | T | C | 0.4508 | 1.72E-08 | 0.08 |
|  | rs17229943 | *OCLN* | 5 | 68682536 | C | A | 0.312 | 1.62E-11 | 0.0463 |
|  | rs385076 | *NLRC4* | 2 | 32489851 | C | T | 0.2432 | 1.66E-22 | 0.0248 |
|  | rs71478720 | *IL10* | 11 | 112009605 | T | C | -0.2669 | 3.71E-22 | 0.0276 |
|  |  |  |  |  |  |  |  |  |  |
| IL-12 | rs7208047 | *RPL7P48* | 17 | 49620242 | G | A | -0.1485 | 3.55E-08 | 0.0269 |
|  |  |  |  |  |  |  |  |  |  |
| IL-23 | rs4921223 | *AC008697.1* | 5 | 158792306  188115682 | G | A | 0.3111 | 8.32E-33 | 0.0261 |
|  | rs9815073 | *LPP* | 3 | 188115682 | A | C | 0.2146 | 8.71E-15 | 0.0277 |
|  |  |  |  |  |  |  |  |  |  |
| IFNγ | rs7459901 | *RP11-756K15.2* | 8 | 58281744 | T | G | 0.3041 | 3.09E-08  4.07E-09 | 0.0549 |
|  | rs7567468 | *UGT1A4* | 2 | 234642838 | T | C | 0.1624 | 4.07E-09 | 0.0276 |

SNP single nucleotide polymorphism, IL interleukin, IFN interferon

Supplementary Table 2: Mendelian randomization estimates for genetically predicted interleukin-18 (standard deviation), based on the 4 genome wide significant SNPs [16, 17], on number of children in the largest available GWAS based on UK Biobank participants of European descent.

| Outcome | Group | Method | # children (standard deviation) | 95% confidence interval | p-value | MR-Egger intercept p-value |
| --- | --- | --- | --- | --- | --- | --- |
| # children fathered | Men | IVW | -0.02 | -0.03 to -0.01 | 0.005 |  |
|  |  | WM | -0.02 | -0.04 to -0.001 | 0.04 |  |
|  |  | MRE | -0.06 | -0.18 to 0.05 | 0.30 | 0.47 |
| # live births | Women | IVW | -0.015 | -0.03 to 0.003 | 0.11 |  |
|  |  | WM | -0.02 | -0.04 to 0.0049 | 0.13 |  |
|  |  | MRE | -0.02 | -0.17 to 0.12 | 0.76 | 0.91 |
|  |  |  |  |  |  |  |
| Overall | All | IVW | -0.018 | -0.029 to -0.007 | 0.001 |  |
|  |  | WM | -0.018 | -0.031 to -0.004 | 0.01 |  |
|  |  | MRE | -0.046 | -0.136 to 0.04 | 0.32 |  |

SNP single nucleotide polymorphism, IVW inverse variance weighted, WM weighted median, MRE MR-Egger

Supplementary Table 3: Mendelian randomization estimates for genetically predicted interleukin-12, interleukin-23 and interferon γ (standard deviations) on different severities of COVID-19 in the largest available GWAS largely of people of European descent compared to the population in the COVID19-hg GWAS meta-analysis round 6 (<https://www.covid19hg.org>)

| Exposure | SNP  # | COVID-19 severity | Method | Odds ratio | 95% confidence interval | p-value |
| --- | --- | --- | --- | --- | --- | --- |
| Interleukin-12 | 1 | Very severe | Wald | 0.97 | 0.73 to 1.30 | 0.86 |
|  |  | Hospitalized | estimate | 0.89 | 0.77 to 1.04 | 0.15 |
|  |  | Any |  | 0.97 | 0.92 to 1.03 | 0.35 |
|  |  |  |  |  |  |  |
| Interleukin-23 | 2 | Very severe | IVW | 1.05 | 0.94 to 1.67 | 0.43 |
|  |  | Hospitalized |  | 0.98 | 0.92 to 1.05 | 0.63 |
|  |  | Any |  | 0.99 | 0.96 to 1.01 | 0.35 |
|  |  |  |  |  |  |  |
| IFNγ | 2 | Very severe | IVW | 1.06 | 0.89 to 1.27 | 0.51 |
|  |  | Hospitalized |  | 1.03 | 0.93 to 1.14 | 0.52 |
|  |  | Any |  | 1.00 | 0.96 to 1.04 | 0.88 |

SNP single nucleotide polymorphism, IVW inverse variance weighted

Supplementary Table 4: Mendelian Randomization estimates for genetically predicted interleukin-18 (standard deviation), based on the 4 genome wide significant SNPs, [16, 17] with parental attained age, in terms of years of life lost, in the largest available GWAS of people of European descent [39]

| Method | Years of life lost | 95% confidence interval | p-value | MR-Egger intercept p-value |
| --- | --- | --- | --- | --- |
| Inverse variance weighting | 0.17 | -0.31 to 0.64 | 0.49 |  |
| Weighted median | 0.05 | -0.51 to 0.61 | 0.85 |  |
| MR-Egger | 1.99 | -2.24 to 6.21 | 0.36 | 0.40 |
